# Mismatch Repair–Independent Increase in Spontaneous Mutagenesis in Yeast Lacking Non-Essential Subunits of DNA Polymerase ε

**DOI:** 10.1371/journal.pgen.1001209

**Published:** 2010-11-18

**Authors:** Anna Aksenova, Kirill Volkov, Jaroslaw Maceluch, Zachary F. Pursell, Igor B. Rogozin, Thomas A. Kunkel, Youri I. Pavlov, Erik Johansson

**Affiliations:** 1 Department of Medical Biochemistry and Biophysics, Umeå University, Umeå, Sweden; 2 Laboratory of Molecular Genetics and Laboratory of Structural Biology, National Institute of Environmental Health Sciences, National Institutes of Heath, Department of Health and Human Services, Research Triangle Park, North Carolina, United States of America; 3 National Center for Biotechnology Information, National Library of Medicine, National Institutes of Health, Bethesda, Maryland, United States of America; 4 Eppley Institute for Research in Cancer, Department of Biochemistry and Molecular Biology, and Department of Microbiology and Pathology, University of Nebraska Medical Center, Omaha, Nebraska, United States of America; Brandeis University, United States of America

## Abstract

Yeast DNA polymerase ε (Pol ε) is a highly accurate and processive enzyme that participates in nuclear DNA replication of the leading strand template. In addition to a large subunit (Pol2) harboring the polymerase and proofreading exonuclease active sites, Pol ε also has one essential subunit (Dpb2) and two smaller, non-essential subunits (Dpb3 and Dpb4) whose functions are not fully understood. To probe the functions of Dpb3 and Dpb4, here we investigate the consequences of their absence on the biochemical properties of Pol ε *in vitro* and on genome stability *in vivo*. The fidelity of DNA synthesis *in vitro* by purified Pol2/Dpb2, i.e. lacking Dpb3 and Dpb4, is comparable to the four-subunit Pol ε holoenzyme. Nonetheless, deletion of *DPB3* and *DPB4* elevates spontaneous frameshift and base substitution rates *in vivo*, to the same extent as the loss of Pol ε proofreading activity in a *pol2-4* strain. In contrast to *pol2-4,* however, the *dpb3Δdpb4Δ* does not lead to a synergistic increase of mutation rates with defects in DNA mismatch repair. The increased mutation rate in *dpb3Δdpb4Δ* strains is partly dependent on *REV3*, as well as the proofreading capacity of Pol δ. Finally, biochemical studies demonstrate that the absence of Dpb3 and Dpb4 destabilizes the interaction between Pol ε and the template DNA during processive DNA synthesis and during processive 3′ to 5′exonucleolytic degradation of DNA. Collectively, these data suggest a model wherein Dpb3 and Dpb4 do not directly influence replication fidelity *per se*, but rather contribute to normal replication fork progression. In their absence, a defective replisome may more frequently leave gaps on the leading strand that are eventually filled by Pol ζ or Pol δ, in a post-replication process that generates errors not corrected by the DNA mismatch repair system.

## Author Summary

The high fidelity of DNA replication is safeguarded by the accuracy of nucleotide selection by DNA polymerases, proofreading activity of the replicative polymerases, and the DNA mismatch repair system. Errors made by replicative polymerases are corrected by mismatch repair, and inactivation of the mismatch repair system results in a multiplicative increase in error rates when combined with a proofreading deficient allele of a replicative polymerase. In this study, we demonstrate that the deletion of two non-essential genes encoding for two subunits of Pol ε give an increased mutation rate due to increased synthesis by the error-prone DNA polymerase ζ. Surprisingly, there was no multiplicative increase in error rates when the mismatch repair system was inactivated. We propose that the deletion of *DPB3* and *DPB4* gives a defective replisome, which in turn gives increased synthesis, in part, by Pol ζ during an error-prone post-replication process that is not efficiently repaired by the mismatch repair system.

## Introduction

The accuracy by which DNA polymerases synthesize DNA is essential for maintaining genome stability and preventing carcinogenesis. Eukaryotes utilize many DNA polymerases, with different properties, during DNA replication and in DNA repair [1]. DNA polymerase δ (Pol δ), DNA polymerase ε (Pol ε) and DNA polymerase α (Pol α) (with associated primase activity) are required for bulk synthesis of DNA during chromosomal replication [2]. Several studies have suggested that there is a division of labor between Pol δ and Pol ε at the replication fork. Genetic and biochemical studies position Pol δ on the lagging strand [3]–[6], whereas Pol ε was shown to participate in the synthesis of the leading strand in *S. cerevisiae* [7]. These studies were preceded by genetic experiments showing that Pol ε and Pol δ proofread opposite strands [8]–[10]. In addition, the Pol ε 3′→ 5′ –exonuclease activity, contrary to the Pol δ 3′→ 5′ –exonuclease activity, does not participate in the correction of errors made by Pol α. This suggests that the proofreading function of Pol ε is restricted to the leading strand [11], while the exonuclease activity of Pol δ, or perhaps another exonuclease, may proofread both strands [12].

The organization of the replication fork during normal DNA replication, with Pol ε on the leading strand and Pol δ on the lagging strand [6], [7], can be disrupted by DNA lesions or sequence contexts in an undamaged template that influence the ability of the replicative polymerase to remain processive [12]–[14]. When polymerases dissociate, the replication machinery must accommodate to complete the replication process and if possible maintain high fidelity. To accomplish this, a variety of strategies are used, including translesion synthesis and recombination pathways [15]. DNA lesions which disengage Pol δ or Pol ε result in single-stranded gaps which are filled in during post-replication repair [16]–[18]. Furthermore, biochemical experiments have shown that collisions between DNA polymerase and transcribing RNA polymerase leads to the abortion of DNA synthesis followed by a reinitiation event when the RNA transcript is used as a primer [19]. To summarize, post-replication repair processes, uncoupled from the replication fork, are likely to occur on both leading and lagging strands to complete DNA replication.

Pol α, Pol δ and Pol ε are all composed of several subunits encoded by separate genes. Besides the catalytic subunit, Pol2 (256 kDa), yeast Pol ε consists of three auxiliary subunits, Dpb2 (79 kDa), Dpb3 (23 kDa) and Dpb4 (22 kDa) [20]. *DPB2* is an essential gene in yeast with an unknown function [21], yet it is required for early steps in DNA replication and is regulated by Cdc28 kinase [22], [23]. Recently *dpb2* mutations that increase spontaneous mutagenesis were found in *S. cerevisiae,* suggesting that the second subunit contributes to the fidelity of DNA replication by an unknown mechanism [24], [25]. *DPB3* and *DPB4* are non-essential genes. Deletion of *DPB3* was previously shown to result in a modest mutator effect [26], [27]. Dpb3 and Dpb4 both contain histone fold motifs that are known to be important in protein-protein interactions [28], [29]. Interestingly, Dpb4 is a component of a chromatin-remodeling complex in *S. cerevisiae*, ISW2, corresponding to the CHRAC complex found in *Drosophila* and humans [30], [31].

The structure of the Pol ε holoenzyme revealed two large domains separated by a flexible hinge [32]. It was suggested that the tail domain of Pol ε was comprised of the Dpb2, Dpb3 and Dpb4 subunits and was important for the binding to and association with the primer-template dsDNA during DNA synthesis [32]. A purified Dpb3-Dpb4 heterodimer was shown to possess dsDNA binding properties, which in part could explain the properties of the tail-domain [29]. However, this does not exclude the possibility that Dpb2 by itself has properties which allow the tail-domain to interact with dsDNA even without Dpb3 and Dpb4.

In this work, we address whether the Dpb3 and Dpb4 subunits have an effect on the biochemical properties of Pol ε and the fidelity of replication in yeast via a function at the tail-domain of Pol ε. We find that Dpb3 and Dpb4 are important for the processivity of Pol ε polymerase and exonuclease activities, suggesting a role of these two subunits in stabilization of Pol ε interaction with primer-template DNA. Evidently this indirectly affects the fidelity of the overall DNA replication process, since deletion of *DPB3* and *DPB4* increases both spontaneous frameshift and base substitution mutagenesis, despite an unchanged fidelity of the purified Pol2/Dpb2 complex. A genetic analysis suggests that *REV3* contributes to the increased mutation rate in *dpb3Δdpb4Δ* and the mutational intermediates escape correction by the mismatch repair system.

## Results

### Influence of *dpb3Δ* and *dpb4Δ* on spontaneous mutagenesis and interaction with *pol2-4*

To investigate the *in vivo* role of the Pol ε accessory subunits Dpb3 and Dpb4, we constructed yeast strains wherein either *DPB3*, *DPB4* or both of these genes were deleted. The frequency of spontaneous mutations in these strains was measured in two reversion assays and one forward mutation assay. We studied the *his7-2* and *lys2::insE-A_14_* reversion alleles to score frameshift mutations. The *his7-2* allele contains a single base pair deletion in a run of 8 T(A) and revert via +1 insertions or -2 deletions [33]. The *lys2::insE-A_14_* allele contains a homonucleotide run of 14 T(A) and revert mainly via -1 mutations [34]. The forward mutation assay scores various types of mutations that inactivate the *CAN1* gene and result in resistance to canavanine. We found that the *dpb3Δ dpb4Δ* double deletion has a moderate mutator effect in all assays. Mutation rates for *his7-2* reversions and *lys2::insE-A_14_* were increased 2.7 and 2.6-fold when compared to the *wt* E134 strain (Table 1). The mutation rate in the forward mutation assay for canavanine resistance was increased 7.4-fold compared to the wt strain (Table 1). The individual contribution of *dpb3Δ* or *dpb4Δ* was comparable to the effect of the deletion of both these genes (*dpb3Δdpb4Δ*) (Table 1). A proofreading deficient allele of the catalytic subunit, *pol2-4*, introduced in the same genetic background resulted in an elevation of the mutation rates similar to the *dpb3Δdpb4Δ* strain (Table 1).

**Table 1:** Spontaneous mutation rates in strains with *dpb3Δ* and *dpb4Δ*, *pol2-4* mutation, *msh6Δ,* and *pol-5DV.*

Strain	Mutation Rate (x10-8) * (95% Confidence Limits)					
	His^+^		Lys^+^		Can^r^	
	Absolute rate	Relative rate (mutants vs. *wt*)	Absolute rate	Relative rate (mutants vs. *wt*)	Absolute rate	Relative rate (mutants vs. *wt*)
*Wild type*	1.5 (0.6–2.4)	1	12.9^a^ § (9.5–26.2)	1	10.1^a^ (3.9–14.6)	1
*dpb3Δ*	4.4 (3.6–5.7)	3.9	35.7 (31.9–56.7)	2.8	81.8 (72.2–95.2)	8.1
*dpb4Δ*	3.7 (3.0–5.0)	2.5	22.3 (19.9–28.6)	1.7	63.4 (57.3–71.4)	6.3
*dpb3Δ dpb4Δ*	4.1 (2.5–5.1)	2.7	33.4§ (25.9–46.4)	2.6	74.3 (66.4–89.5)	7.4
*pol2-4*	6.9 (4.2–8.6)	4.6	35.7§ (32.9–48.3)	2.7	77.9 (69.4–90.5)	7.7
*pol2-4 dpb3Δ*	13.4 (10.9–18.8)	8.9	44.5 (37.8–52.5)	3.4	153 (128–210)	15.1
*pol2-4 dpb4Δ*	28.1 (23.0–30.4)	18.7	44.5 (39.1–59.9)	3.4	224 (200–268)	22.2
*pol2-4 dpb3Δ dpb4Δ*	22.1 (18.5–26.8)	14.8	41.0§ (35.6–49.3)	3.2	132 (123–170)	13
*msh6Δ*	5.3 (3.5–6.7)	3.5	2720 (2390–3690)	211	165 (119–226)	16.3
*msh6Δ dpb3Δ*	7.0 (5.5–9.4)	4.6	1360 (1100–1550)	105	299 (216–399)	29.6
*msh6Δ dpb4Δ*	7.6 (6.3–9.3)	5.1	1430 (1180–1590)	111	278 (217–344)	27.5
*msh6Δ dpb3Δ dpb4Δ*	9.0 (8.1–12.4)	6.0	2200 (2060–2830)	171	261 (217–346)	25.8
*msh6Δpol2-4*	71.1 (55.7–82.7)	47.4	7180 (6190–8870)	557	7150 (6410–9810)	708
*msh6Δ pol2-4 dpb3Δ*	72.6 (45.9–119)	48.4	2460 (1800–4670)	190	13400 (11200–17000)	1320
*msh6Δ pol2-4 dpb4Δ*	76.6 (32.6–121)	51.1	2590 (1870–5510)	201	17800 (10500–21100)	1760
*msh6Δ pol2-4 dpb3Δ dpb4Δ*	68.6 (54.9–100)	45.7	2600 (594–4010)	201	8450 (6670–13800)	837
*pol3-5DV*	7.3 (6.2–8.8)	4.9	71 (57–77)	5.5	350 (304–419)	35
*pol3-5DV dpb3Δ dpb4Δ*	17.8 (15–21)	11.9	67 (54–85)	5.2	363 (288–547)	36

The genetic experiments were performed with derivatives of the strain created by [34] and named E134 [33] obtained as described in Materials and Methods.

^a^Mutation rates are given as median of one experiment with nine independent cultures and coincide with previously published data.

*: For all other cases, mutation rates were obtained as the median of 18–45 independent cultures and determined as described in [33]. In all cases, the mutation rates in mutants differ from that in the wild type (confidence limits do not overlap).

**§:** 9/9 sequenced revertants contained -1 frameshift mutation within 14A run.

To determine if the participation of Pol ε in DNA replication depends on *DPB3* and *DPB4,* we combined *dpb3Δ*, *dpb4Δ*, or *dpb3Δ dpb4Δ* with the *pol2-4* mutation. The analysis revealed different genetic interactions. Combining *dpb3Δ* and *pol2-4* led to an additive effect on *his7-2* reversion (Table 1). A higher than additive increase in mutation rate was observed with the *his7-2* allele when *pol2-4* was combined with *dpb4Δ* or *dpb3Δ dpb4Δ* (Table 1). Reversions scored in the *lys2::insE-A_14_* allele revealed a close to epistatic interaction between *pol2-4, dpb3Δ, dpb4Δ,* and *dpb3Δ dpb4Δ* (Table 1). The *pol2-4* mutation itself elevated the reversion rate of the *lys2::insE-A_14_* allele 2.7-fold, which agrees with previous results [35], [36]. The forward mutation assay with the *CAN1* gene revealed an additive effect of the *pol2-4* mutation and the double *dpb3Δ dpb4Δ* deletion. An additive interaction was also found in the *pol2-4dpb3Δ* strain, but the combination of *pol2-4* and *dpb4Δ* gave a higher than additive increase in mutation frequency (Table 1). The disparate genetic interactions of *DPB3* and *DPB4* with the proofreading activity of Pol2 could be due to the separate function of Dpb4 in a chromatin remodeling complex, ISW2 [30], [31], [37], [38]. However, there are no reports demonstrating that ISW2 influence the mutation rate in *S. cerevisiae*. Another possibility could be that Dpb3 and Dpb4 influence the fidelity of DNA synthesis by Pol ε.

### Fidelity of Pol2/Dpb2 *in vitro*

To measure the fidelity of Pol ε lacking Dpb3/Dpb4, we purified the wild type (i.e., exonuclease proficient) Pol2/Dpb2 complex and the exonuclease deficient pol2-4/Dpb2 complex, and then measured their fidelity in an M13mp2 gap-filling assay [39]. The *lacZ* mutant frequency of the DNA synthesis reaction products generated by the wild type Pol2/Dpb2 complex was 0.0018, comparable to the previously reported value of 0.0019 for the four-subunit Pol ε [40]. Both values are near the background *lacZ* mutant frequency of uncopied DNA, indicating that the exonuclease proficient Pol2/Dpb2 complex is highly accurate. The pol2-4/Dpb2 complex was less accurate, as expected because it is proofreading deficient. However, it was no less accurate than the exonuclease-deficient 4-subunit holoenzyme, as indicated by the similar *lacZ* mutant frequencies observed for both complexes (Table 2). To analyze if the error specificity of the 2-subunit enzyme differed from that of the holoenzyme, we sequenced 277 independent mutants generated by pol2-4/Dpb2, and compared the results to those reported in an earlier study [40] of 285 *lacZ* mutants generated by the holoenzyme. Comparable error specificity was observed (Table 2) for substitutions, frameshifts and other mutations. We conclude that the increased mutation rates in the *dpb3Δ dpb4Δ* strain is unlikely to be due to a lower fidelity of DNA synthesis by Pol ε *per se*.

**Table 2:** Mutations generated by exonuclease-deficient Pol ε *in vitro*.

Polymerase	Holoenzyme (pol2-4)	pol2-4/Dpb2
Mutant Frequency	0.026	0.029
Total Mutants Sequenced	285	277
Substitutions	214	229
−1 frameshifts	53	35
+1 frameshifts	9	7
Other mutations^a^	11	29

The results for the holoenzyme are from [40].

For both enzymes, only phenotypically detectable changes in the *lacZ* gene are included.

a Other mutations include deletions of 2–3 bases, more complex substitution-deletions, and deletions of larger numbers of bases between direct repeat sequences. Statistical analysis of the distributions of substitutions produced by the four subunit and two-subunit pol ε along lacZ was performed using the COLLAPSE program [70].These two spectra are not different (P = 0.90). This result strongly suggest that properties of four subunit and two subunit polymerases are highly similar (linear correlation coefficient for the two spectra  = 0.72, P<0.01). We also compared the raw data from Table 2 using the same approach. These two distributions are different (P = 0.005). However, they are not different after the removal of the category “Other mutations” (P = 0.11). The only reason why two spectra are different are long deletions (>100 bp) that are included under “Other mutations.” After removal of these long deletions the spectra are not different (P = 0.17).

### Genetic interaction between *rev3Δ* and *dpb3Δ dpb4Δ*

The *REV3* gene encodes the catalytic subunit of DNA polymerase ζ (Pol ζ), which is known to be a major contributor to both spontaneous and DNA damage inducible mutagenesis in wild type strains and in strains with defects in other DNA polymerases [27], [41], [42]. Yeast Pol ζ has relatively high fidelity for single-base insertions and deletions, and somewhat lower fidelity for base substitutions [43]. Deletion of the *REV3* gene suppresses mutagenesis in *CAN1* in the *dpb3Δ dpb4Δ* strain but not mutagenesis in the *his7-2* or *lys2::insE-A_14_* allele (Table 3). Thus, the increase in frameshift mutagenesis observed in the *his7-2* and *lys2::insE-A_14_* alleles is Pol ζ-independent.The independence of frameshift *his7-2* and *lys2::insE-A_14_* reversion from Pol ζ is consistent with an earlier observation that replication defects (e.g. in Pol δ mutant, the *pol3-Y708*allele) cause Pol ζ dependent mutagenesis for base substitutions only [44].

**Table 3:** Influence of *rev3Δ* on spontaneous mutagenesis in the strain lacking *DPB3* and *DPB4* genes.

Strain	Mutation Rate (x10-8) * (95% Confidence Limits)					
	His^+^		Lys^+^		Can^r^	
	Abs.^a^	Rel.^b^	Abs.	Rel.	Abs.	Rel.
*dpb3Δ dpb4Δ*	4.1 (2.5–5.1)	1	33.4 (25.9–46.4)	1	74.3 (66.4–89.5)	1
*dpb3Δ dpb4Δ rev3Δ*	3.6 (2.7–4.2)	0.9	33.2 (30.1–39.1)	1	26^c^ (20.7–29.1)	0.3

The genetic experiments were performed with derivatives of the strain created by [34] and named E134 [33] obtained as described in Materials and Methods.

*Mutation rates were obtained as median of 18–45 independent cultures and determined as described in [33].

a Absolute mutation rate for a particular mutation event.

b Relative mutation rate – mutation rate for a particular strain vs. mutation rate for *Wild type.*

c There was only a significant difference for *CAN1* when comparing mutation rates in *REV3* and *rev3Δ* strains.

### Genetic interaction between Pol δ and *dpb3Δ dpb4Δ*

Published results suggest that Pol δ can proofread errors made by Pol α [11]. To ask if Pol δ proofreads errors generated in the *dpb3Δ dpb4Δ* strain, we combined the proofreading deficient Pol δ allele *pol3-5DV* with *dpb3Δ dpb4Δ*. The *pol3-5DV dpb3Δ dpb4Δ* strain was viable, in contrast to *pol3-01 pol2-4* and *pol3-5DV pol2-4* haploid strains [9]. The mutation rates in *pol3-5DV dpb3Δ dpb4Δ* and *pol3-5DV* in the *CAN1* gene were similar (Table 1). In contrast, the reversion rate of the *his7-2* allele was greater than additive in the *pol3-5DV dpb3Δ dpb4Δ* strain, when compared to the *pol3-5DV* strain and *dpb3Δ dpb4Δ* strain. We conclude that Pol δ has the capacity to proofread a fraction of frameshift errors that occur in the *dpb3Δ dpb4Δ* strain, but there could also be some other 3′→5′ exonuclease that participates in the process.

### Genetic interaction with *MSH6*, *MSH2*, *MLH1*, and *PMS1*

The mismatch repair protein Msh6 is involved in recognizing a subset of replication errors, specifically single base mismatches and small insertion-deletion intermediates [45]. Although less severe than *msh2Δ*, *pms1Δ* or *mlh1Δ*, inactivation of the *MSH6* gene results in a strong increase in mutagenesis (Table 4). For instance, *msh6Δ* leads to a dramatic increase of *lys2::insE-A14* allele reversion rates as a result of single nucleotide deletions (Table 4, [34], [35]). To ask whether *DPB3* and *DPB4* interact with the mismatch repair system we measured the mutation rates in strains with *dpb3Δ* and *dpb4Δ* deletions in an Msh6-deficient background to score for base-base mismatches and small insertion-deletion errors.

**Table 4:** Spontaneous mutation rates in strains with *dpb3Δ dpb4Δ* and mismatch repair deficiency.

Strain	Mutation Rate (x10-8) * (95% Confidence Limits)					
	His^+^		Lys^+^		Can^r^	
	Absolute rate	Relative rate (mutants vs. *wt*)	Absolute rate	Relative rate (mutants vs. *wt*)	Absolute rate	Relative rate (mutants vs. *wt*)
*Wild type*	1.5 (0.6–2.4)	1	12.9^a^ § (9.5–26.2)	1	10.1^a^ (3.9–14.6)	1
*dpb3Δ dpb4Δ*	4.1 (2.5–5.1)	2.7	33.4§ (25.9–46.4)	2.6	74.3 (66.4–89.5)	7.4
*msh6Δ*	5.3 (3.5–6.7)	3.5	2720 (2389–3690)	211	165 (119–226)	16.3
*msh6Δ dpb3Δ dpb4Δ*	9.0 (8.1–12.4)	6.0	2203 (2061–2832)	171	261 (217–346)	25.8
*mlh1Δ*	120 (101–144)	80	126060 (110000–161000	9770	772 (506–895)	76
*mlh1Δdpb3Δ dpb4Δ*	103 (87–110)	69	136955 (116000–144000)	10600	505 (429–669)	50
*pms1Δ*	68 (53–84)	45	123499 (110000–155000)	9570	449 (346–666)	44
*pms1Δ dpb3Δ dpb4Δ*	88 (62–126)	59	128931 (101000–148000)	9990	446 (374–697)	44
*msh2Δ*	69 (52–86)	46	126181 (85000–154000)	9780	418 (355–671)	41
*msh2Δ dpb3Δ dpb4Δ*	115 (95–149)	77	168424 (134000–316000)	13100	662 (512–835)	66

The genetic experiments were performed with derivatives of the strain created by [34] and named E134 [33] obtained as described in Materials and Methods.

^a^Mutation rates are given as median of one experiment with nine independent cultures and coincide with previously published data.

*For all other cases mutation rates were obtained as median of 18–45 independent cultures and determined as described in [33].

**§:** 9/9 sequenced revertants contained -1 frameshift mutation within 14A run.

The combination of the *msh6Δ* with the *dpb3Δ* and *dpb4Δ* gave an additive increase in the *his7-2* reversion and *can1* mutation rates (Table 1). The strong synergetic interaction between proofreading defects (*pol2-4*, *pol3-01*) and defects in the mismatch repair system was previously observed for short homopolymeric runs and base substitutions, but not for long homopolymeric runs, such as the A14 run in the *lys2::insE-A_14_* allele [34], [35], [46]. In agreement with that, we observed a synergetic interaction between *pol2-4* and *msh6Δ* when mutation rates in a *pol2-4 msh6Δ* strain were estimated in the *his7-2* and *CAN1* loci (Table 1). In the short 8A run of the *his7-2* allele, neither the deletion of *DPB3* or *DPB4* nor both genes affected the mutation rate of the *pol2-4 msh6Δ*. The multiplicative interaction of *pol2-4* and *dpb4Δ* is absent in the *msh6Δ* background. The mutation rate in the *lys2::insE-A_14_* gave a complex interaction between *msh6Δ* and *dpb3Δ* and *dpb4Δ*. The mutation rate was somewhat lower (though not statistically significant, see overlapping confidence limits in Table 1) when either *dpb3Δ* or *dpb4Δ* was combined with *msh6Δ*, than when the *dpb3Δ dpb4Δ* was combined with *msh6Δ*. When *pol2-4* was added to the *msh6Δ* strain, the combination with *dpb3Δ, dpb4Δ* or *dpb3Δ dpb4Δ* gave a mutation rate that was one third of the mutation rate in the *pol2-4 msh6Δ* strain. At present, it is not clear why this small reduction in mutation rate occurs.

The lack of a synergetic interaction between *dpb3Δ*, *dpb4Δ* and *msh6Δ* was unexpected and led us to ask if this was also true for other genes that are required for mismatch repair. Msh2 forms a heterodimer with either Msh3 or Msh6. Thus, *msh6Δ* strains still have active Msh2-Msh3 which corrects most replication errors. To completely abolish mismatch repair we deleted *MSH2, MLH1*, or *PMS1*. The combination of *mlh1Δ* or *pms1Δ* with *dpb3Δ dpb4Δ* did not reveal a strong synergetic interaction on the *his7-2* reversion or *can1* mutation rates (Table 4). The combination of *msh2Δ* and *dpb3Δ dpb4Δ* gave only a two-fold increase in mutation rate on *his7-2* reversions and no increase on *can1* mutation rates. These data indicate that *dpb3Δ dpb4Δ* do not act in series with the mismatch repair system.

### DNA sequence changes in the *CAN1* gene in strains lacking *DPB3* and *DPB4* genes

Forward mutations giving resistance to canavanine can arise by many different mechanisms. Earlier studies have shown that even a small collection of sequenced *can1* mutants can reveal significant changes in the mutation spectra (e.g. upon deletion of *POL32*, *a* small subunit of Pol δ, or inactivation of Pol ε proofreading with the *pol2-4* allele [9], [47]). To analyze if the deletion of *DPB3* and *DPB4* might drastically influence the distribution of types of mutations in the *CAN1* gene, we sequenced 48 *can1* mutants in four isogenic strains: *wt*, *dpb3Δdpb4Δ*, *pol2-4* and *pol2-4 dpb3Δdpb4Δ* (Table 5). The difference between strains carrying the *pol2-4* allele and carrying *POL2* was statistically significant according to a modified Pearson χ^2^ test of spectra homogeneity (see Table S1 and Table S2). There was a characteristic reduction of CG→GC changes and an increase of frameshift mutations in the *pol2-4* spectrum (Table 5). The comparison between *wt* and *dpb3Δ dpb4Δ* or *pol2-4* and *pol2-4 dpb3Δ dpb4Δ* showed that the deletion of *DPB3* and *DPB4* did not give a statistically significant alteration in mutation spectra (see Table S1). The sample size was insufficient to demonstrate the enhancement of an error signature for Pol ζ despite the increased contribution of *REV3* dependent mutations in *CAN1*. We conclude that the *CAN1* mutations appearing in the *pol2-4* background and in the *dpb3Δ dpb4Δ* mutants arise by different mechanisms.

**Table 5:** *CAN1* forward mutation spectrum.

Types of mutations	Amount of mutations in strains:			
	Wild type	*dpb3Δ dpb4Δ*	*pol2-4*	*pol2-4dpb3Δ dpb4Δ*
Base substitutions:				
AT->TA	1 (2.1%)	1 (2.0%)	6 (12.5%)	6 (12.5%)
AT->CG	2 (4.2%)	1 (2.0%)	5 (10.4%)	4 (8.3%)
AT->GC	3 (6.3%)	3 (6.1%)	5 (10.4%)	0 (0.0%)
GC->CG	12 (25.0%)	9 (18.4%)	4 (8.3%)	8 (16.7%)
GC->AT	7 (14.6%)	9 (18.4%)	3 (6.3%)	2 (4.2%)
GC->TA	10 (20.8%)	8 (18.3%)	6 (12.5%)	7 (14.6%)
-1 frameshifts	5 (10.4%)	9 (18.4%)	5 (10.4%)	9 (18.8%)
+1 frameshifts	2 (4.2%)	1 (2.0%)	11 (22.9%)	6 (12.5%)
Complex	1 (2.1%)	5 (10.2%)	2 (4.2%)	3 (6.3%)
Other	5 (10.4%)	3 (6.1%)	1 (2.1%)	3 (6.3%)
Total	48 (100%)	49^a^ (100%)	48 (100%)	48 (100%)

a One of the sequenced Can^r^ genes carried two independent mutations located 215 nt apart.

### Dpb3 and Dpb4 subunits are required for both processive polymerase and processive 3′ to 5′ exonuclease activity of Pol ε

**Figure 1:**
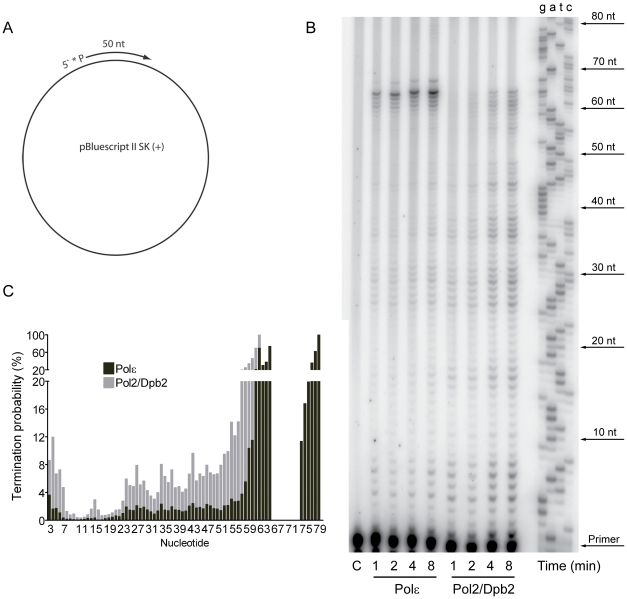
Processivity of the polymerase activity of Pol ε holoenzyme and Pol2/Dpb2 complex. (A) A 50 nt long, ^32^P-5′end-labeled, oligonucleotide was annealed to pBluescript II SK (+) ssDNA and used as a DNA substrate in the polymerization assay. (B) Shown is the image of extension products generated by four-subunit Pol ε and a two-subunit Pol2/Dpb2 complex, separated on a 8% denaturing polyacrylamide gel (for details see Materials and Methods). A DNA sequencing ladder with the identical template was used as a molecular weight marker on the right hand. Reaction times are indicated under each lane. (C) The termination probability at each position on the template was calculated for the four-subunit Pol ε holoenzyme and Pol2/Dpb2 complex (for details see Materials and Methods).

The contribution of Pol ζ to the elevated mutation rates suggested that a Pol2/Dpb2 complex does not support a fully functional replisome. To ask if Dpb3 and Dpb4 influence the processivity of Pol ε, we purified a Pol2/Dpb2 complex with an intact polymerase and exonuclease activity. We measured the processivity of the polymerase activity on a singly-primed, single stranded circular DNA template under single-hit conditions [48]. We found that a 40-fold molar excess of the primer-template over Pol ε and a 20-fold molar excess of the primer-template over Pol2/Dpb2 fit the criteria for single-hit conditions. The processivity of Pol ε on this template was comparable to a previous report, with a strong pause-site 63 nucleotides from the primer (Figure 1B) [48]. The absence of Dpb3 and Dpb4 from Pol ε lowers the processivity of polymerization. Some products reached a length of 63 nucleotides, but products were terminated with a higher probability at numerous positions (Figure 1C). On average, the termination probability at each position on the template increased two to three-fold for Pol2/Dpb2 as compared to the four subunit Pol ε.

**Figure 2:**
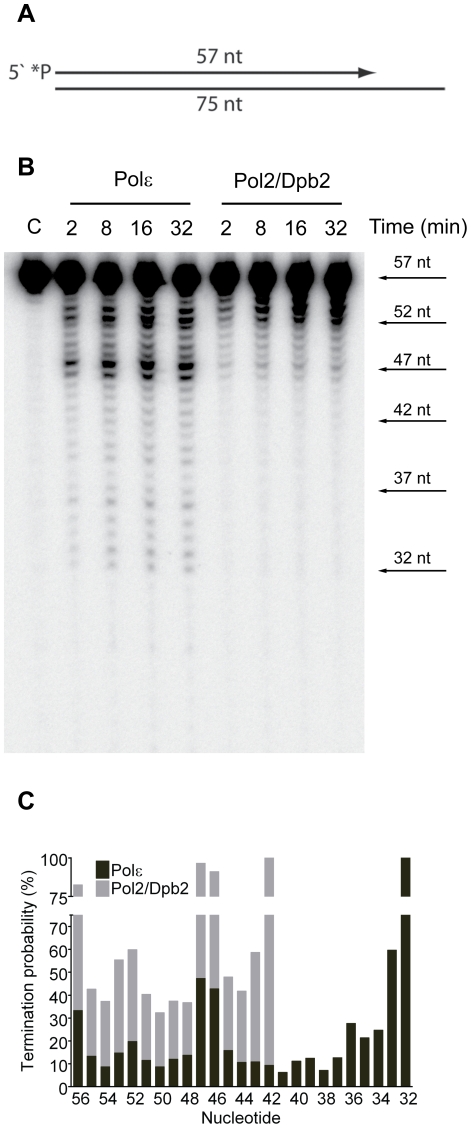
Processivity of the exonuclease activity of Pol ε holoenzyme and Pol2/Dpb2 complex. (A) A 57 nt long, ^32^P-5′end-labeled, oligonucleotide was annealed to a 75 nt oligonucleotide, creating a primer-template to be used as a DNA substrate in the exonuclease assay. (B) Shown is the image of degradation products generated by four-subunit Pol ε and two-subunit Pol2/Dpb2 complex, separated on a 12% denaturing polyacrylamide gel (for details see Materials and Methods). Reaction times are indicated above each lane. (C) The termination probability at each position on the template was calculated for the four-subunit Pol ε holoenzyme and Pol2/Dpb2 complex (for details see Materials and Methods).

Next, we asked if the Dpb3 and Dpb4 subunits are required for processive exonucleolytic degradation of DNA. We carried out an exonuclease assay with a 57-nt long primer annealed to a 75-nt long template to generate 57-nt dsDNA region. Again, the conditions were empirically determined to achieve single-hit kinetics. This time a five-fold molar excess of primer-template over the four-subunit Pol ε was used, whereas an equimolar ratio of primer-template and enzyme was used for Pol2/Dpb2. We found that the Pol ε holoenzyme efficiently degraded the first 24 nucleotides of the primer (Figure 2B). At this point only ∼32 nt of the primer remained. This correlates well with the minimal length of dsDNA required for processive synthesis of DNA by Pol ε [32]. By analogy, the processivity of Pol ε exonuclease activity could depend on a specific interaction between the tail-domain and the dsDNA. In agreement with this hypothesis, we found the exonuclease activity of Pol2/Dpb2 to be less processive. Very few primers were degraded further than 11 nucleotides. On average, the termination probability at each position on the primer increased two to three-fold for Pol2/Dpb2 as compared to four-subunit Pol ε (Figure 2C). In addition, the absence of Dpb3 and Dpb4 did not result in a general inactivation of the exonuclease activity, since the exonuclease activity of the Pol2/Dpb2 complex and four-subunit Pol ε was comparable on single-stranded DNA (data not shown). We conclude that Dpb3 and Dpb4 stabilize the interaction of Pol ε with primer-template DNA and therefore positively affect the processivity of the polymerase and exonuclease activities of Pol ε. The removal of Dpb3 and Dpb4 would then lead to frequent dissociation of Pol ε that may disrupt the synthesis of the leading strand and potentially result in single-strand gaps.

## Discussion

In general, defects at the replication fork which give higher mutation rates act in series when combined with an inactivated mismatch repair system, i.e. mutator alleles of the catalytic subunit of Pol α, Pol δ, and Pol ε, temperature sensitive mutations of *DPB2* (subunit of Pol ε), *rfa1-29t*, or the *rfc1::Tn3* allele (subunit of clamp loader) [9], [24], [25], [49]–[52]. The interpretation has been that errors made in the proximity of the replication fork are corrected by mismatch repair and this results in a synergistic increase in mutation rates when mismatch repair is inactivated. In this study we show that deletion of *DPB3* and *DPB4* have the unique property among replication fork associated genes to give an increased mutation rate, but *do not* act in series with the mismatch repair system.

### Defective replisome-driven mutagenesis

The unaltered fidelity of the Pol2/Dpb2 complex suggested that Dpb3 and Dpb4 are not important for the fidelity of DNA synthesis by Pol ε per se. In contrast, our genetic analysis demonstrated that the inactivation of *DPB3* and *DPB4* in yeast elevates the mutation rates comparable to the proofreading deficient *pol2-4* allele of Pol ε. This suggests that the dynamics of the replication fork was altered in the *dpb3Δ dpb4Δ* strain and the defect influenced the fidelity of the replisome. The hypothesis was supported by the observation that a Pol2/Dpb2 complex (lacking Dpb3 and Dpb4) was less processive both when polymerizing new DNA and degrading DNA (Figure 1 and Figure 2).

Recently, it was shown that Pol ζ participates in the synthesis on undamaged DNA templates during defective replisome-induced mutagenesis as well as synthesis on stretches of single-stranded DNA carrying DNA lesions [42], [53]. Our genetic analysis supports a role for Pol δ and Pol ζ in spontaneous mutagenesis in *dpb3Δ dpb4Δ* strains since the mutagenesis in *CAN1* depends in part on Pol ζ and Pol δ proofreading suppresses mutations in *his7-2*. One explanation for our observations could be that Pol ε dissociates more frequently from the template when *DPB3* and *DPB4* are deleted. After reinitiation, a gap is left that will be filled in by a post-replication repair mechanism analogous to what might happen when a replicative polymerase encounters a DNA lesion that cannot be bypassed. During this process, there will be time for the 3′-end to repeatedly melt and reanneal. A short homonucleotide run at the *his7-2* site may frequently reanneal at the wrong nucleotide creating 1 or 2 nt loops. Such errors could be corrected by proofreading by the replicative polymerases [34] and the presence of Exo+ Pol δ during the gap-filling process would lead to decreased level of mutations. In a *pol3-5DV* strain, this proofreading is absent leading to a more than additive increase in mutation rate. In this scenario, we detect errors that appear not because of synthetic errors by Pol ε but instead due to an intermediate DNA structure that is prone to frameshift mutations. Thus a greater than additive interaction could be expected because Pol ε without Dpb3 and Dpb4 and proofreading by Pol δ act in series. The effect is detected because of the property of the reversion assay, which focuses on a single mutational pathway. We do not observe the same effects in the *CAN1* gene because, in this case, we detect mutations generated by many different pathways. There is, however, a strong synergetic interaction between *pol3-5DV* and inactivation of mismatch repair. This can easily be explained by the proofreading deficiency of *pol3-5DV* that generates errors on the lagging strand at the replication fork. In addition, *pol3-5DV* is a mutator allele due to a defect in Okazaki fragment maturation [54]. Because of the multiple roles of Pol δ and its proofreading activity, more experiments are required to establish the nature of the effect of the *pol3-5DV* that we observed for *his7-2* reversion.

The deletion of *DPB3* and *DPB4* could also result in lesser overall DNA synthesis by Pol ε on the leading strand. This is not likely to be the case as the mutation rate in the *pol2-4* strain is not higher than in the *pol2-4 dpb3Δ dpb4Δ* strain and the mutation signature from *pol2-4* in the *CAN1* gene is also found in the *pol2-4 dpb3Δ dpb4Δ* strain, suggesting that exonuclease deficient pol2 synthesize approximately the same amount of DNA regardless if Dpb3 and Dpb4 are present or not.

It was earlier shown that defective replicative DNA polymerases (encoded by *pol1-1, pol2-1, pol3t and pol3-Y708A*) lead to an increased mutation rate that is in part dependent on Pol ζ. To our knowledge, the *in vitro* fidelity of the enzymes encoded by the four mutant alleles, *pol1-1*, *pol2-1*, *pol3t* and *pol3-Y708A* has not been measured. Thus, it is not firmly established if these alleles replicate DNA with a reduced fidelity. It is however, plausible that pol3-Y708A has a reduced fidelity based on analogous mutations in the Klenow fragment and RB69 DNA polymerase (discussed in [44]) and the position of Tyr708 in the active site [55]. The *pol3-t* mutant has a temperature sensitive mutation that also may affect the polymerase site and alter the fidelity of Pol3 [44], [55]. In cases where the effect of mismatch repair has been studied, clear synergy was observed for *pol1-1* [56], *pol3-t* [57] and *pol3-Y708A* [44]. Mutant alleles encoding for polymerases that by itself synthesize DNA with a higher error-rate are likely to show synergy with the inactivation of mismatch repair, even if a substantial part of the mutations in the strain are *REV3* dependent. The dual mechanism of mutator effects are exemplified by *pol3-Y708A*, which is likely to encode a polymerase that both generate errors that are corrected by mismatch repair and also induce PCNA ubiquitylation and a Pol ζ dependent increase of mutation rates. Here we present, for the first time, data on a mutant possessing two-subunit Pol ε with a confirmed unchanged error-rate *in vitro*, and a Pol ζ dependent increase in mutation rates, but no observed synergy with the inactivation of mismatch repair. This observation provides a distinction of errors made at the replication fork from errors made during postreplication DNA synthesis.

### Errors depending on *dpb3*Δ *dpb4*Δ are not corrected by mismatch repair system

The deletions of *DPB3* and *DPB4* led to an increased mutation rate but did not act in series with *msh6*Δ, *msh2*Δ, *pms1*Δ or *mlh1*Δ. The lack of synergy could be due to an essential function for *DPB3* or *DPB4* in mismatch repair that inactivates the mismatch repair system. It was proposed earlier that the 3′→ 5′exonuclease activity of Pol ε could be involved in the excision step of mismatch repair in yeast [35]. However, the reversion rate at the *lys2::insE-A14* allele in the *dpb3Δ dpb4Δ* strain is too low to support a role for *DPB3* and *DPB4* in mismatch repair (compare *dpb3Δ dpb4Δ* with *msh6*Δ, *msh2*Δ, *pms1*Δ or *mlh1*Δ (Table 1 and Table 4)). Yet, there is a possibility that redundancy, due to genes with over-lapping functions in mismatch repair, suppress the mutation rate in *dpb3*Δ *dpb4*Δ strains. The possible redundancy only allows us to conclude that there is no evidence for a role of *DPB3* and *DPB4* (or Pol ε) in mismatch repair.

Whether mismatch repair is carried out in the near proximity of the replication fork or is uncoupled from the replication fork remains unclear. It has been proposed that mismatch repair may be physically linked to the replication fork [58], but DNA lesions from MNNG may induce a futile repair cycle where mismatch repair functions outside the S-phase [59]. Based on the genetic analysis of *DPB3* and *DPB4* we propose a model with two zones where mutagenesis occurs during DNA replication. The first zone is in the near proximity of the replication fork where Pol ζ- independent mutagenesis occurs and errors are corrected by mismatch repair. The second zone where Pol Δ and Pol ζ carries out post-replication repair is uncoupled from the replication fork. In this zone, Pol ζ-dependent mutagenesis occurs and errors are not at all or very inefficiently corrected by the mismatch repair system.

There is a series of observations upon which this model is based. We have clearly shown that the mutagenesis of the *CAN1* gene in *dpb3*Δ *dpb4*Δ strains depends on Pol ζ and some mutation intermediates in the *his7-2* allele are proofread by Pol Δ. Gap-filling during a post-replication repair process is likely to depend on PCNA, thus giving an advantage to Pol δ and Pol ζ over Pol ε to synthesize DNA since Pol ε has a slow on-rate on the PCNA-primer ternary structure [14], [48]. Recently, during defective-replisome-induced mutagenesis it was independently shown that Pol ζ replicates undamaged DNA under conditions when the dynamics of the replication fork is affected [42]. Mismatch repair is functional in the *dpb3*Δ *dpb4*Δ background and yet there is no synergism. A hypothetical role for Rev3 in the close proximity of the replication fork would result in replication errors that are expected to be corrected by the mismatch repair system, analogous to errors produced by proofreading deficient Pol ε. At this position Rev3 would be a major contributor to the mutation rate in a *msh2*Δ strain, because Rev3 is responsible for at least half the mutation rate in the *CAN1* gene in wild-type strains. The combination of *rev3*Δ and *msh2*Δ would then result in a substantially lower mutation rate; however, this was not the case. Instead, the mutation rate in a *rev3*Δ*msh2*Δ strain was comparable to a *msh2*Δ strain [47], suggesting that errors by Pol ζ are not efficiently corrected by mismatch repair. Our results demonstrate that errors generated by Pol ζ are not efficiently repaired by mismatch repair and are supported by evidence that some mutations generated by Pol ζ in a *rad52*Δ background are not corrected by mismatch repair in the *lys2*Δ *A746-NR* allele [60]. Based on the sum of these observations we propose that the deletion of *DPB3* and *DPB4* results in a decreased Pol ε processivity, generating a DNA substrate, which must be processed to some extent by Pol δ and Pol ζ during post-replication repair. This event occurs in a zone separated from active replication forks where the correction of errors by mismatch repair may be inefficient.

Alternative interpretations could be that synthesis by Pol ζ at stalled replication forks is not under mismatch repair surveillance. The transient dissociation of Pol ε would, in this case, create specific conditions when mismatch repair cannot function. Under these specific conditions any repair synthesis at the replication fork as well as post replicative repair synthesis in the single stranded gaps might escape MMR. The influence of chromatin on mutation rates and mismatch repair could also be an explanation. A potential mechanism could be that PCNA is post-translationally modified, due to check-point activation, blocking the interaction between mismatch repair proteins and PCNA. Regions with post-translationally modified PCNA would then be less efficiently repaired by mismatch repair.

Mismatch repair genes are not exclusively involved in correcting replication errors at the replication fork. Recently, it was shown that mismatch repair genes suppress recombination and promote translesion synthesis by Pol ζ in an assay measuring spontaneous mutation rates [60]. Other examples are immunoglobulin genes where mismatch repair together with Pol η is required for hypermutation at A/T pairs [61]. This is a paradox as the mismatch repair system promotes error-prone DNA synthesis by Pol ζ and Pol η in these two examples. The deletion of *DPB3* and *DPB4* unveils another example of how error-prone DNA synthesis is accepted to complete DNA synthesis and the mismatch-repair system does not correct the errors. The contribution of these Pol ζ dependent errors is small when compared to the error load which is corrected by mismatch repair at the proximity of the replication fork (compare *CAN1* mutation rate in *dpb3Δ dpb4Δ* with *msh2*Δ (Table 4)). Yet, we found that the error-rate in the *dpb3*Δ *dpb4*Δ strain was comparable to the *pol2-4* strain. The error-rate in *pol2-4/pol2-4* mice was recently reported to be sufficient to support tumor development in mice [62]. Although the mechanism by which the error rates increases in *pol2-4* and *dpb3Δ dpb4Δ* strains clearly differs, it is tempting to speculate that the inactivation of the mammalian homologues to *DPB3* and *DPB4* could result in defective replisomes, elevated mutation rates and tumor development.

## Materials and Methods

### Yeast strains

All *S. cerevisiae* strains used in this study are isogenic to E134 (*MATα ade5-1 lys2::InsEA14 trp1-289 his7-2 leu2-3,112 ura3-52*) [33]. The *dpb3Δ* mutant was kindly provided by P. Shcherbakova and is described in [27]. Other strains carrying *dpb3Δ* were obtained as described in [27]. The *dpb4Δ* mutants were constructed by transformation with PCR fragment carrying the *hygB* selectable marker and obtained using primers DPB4/kanMX-F (5′-ATGCCACCAAAAGGTTGGAGAAAAGACGCCCAAGGGAATTACCCCCGTACGCTGCAGGTCGAC) and DPB4/kanMX-R (5′-TTACGTTTGCTCAAGGTTTTGAACTCTAGTTTCTACATCTTGGCTATCGATGAATTCGAGCTCG) and the pAG32 plasmid as a template [63]. The disruption was confirmed by PCR analysis. The *pol2-4* mutation was obtained as described in [64] using YIpJB1 plasmid carrying *pol2-4* mutation [65]. The *pol3-5DV* mutation was obtained as described in [64] using plasmid p170-5DV [66].The presence of the *pol2-4* mutation after integration into the chromosome was confirmed by SfcI digest of short PCR fragment encompassing mutation and DNA sequencing. Deletion of the *MSH6* gene was obtained as described in [67]. The *REV3* gene, encoding the catalytic subunit of Pol ζ, was deleted as described in [68]. Deletion of the *MSH2* gene was obtained by transformation with PCR product obtained from the pRS305 plasmid using oligos MSH2_del_F (CTCCACTAGGCCAGAGCTAAAATTCTCTGATGTATCAGAGGAGAGCAGAGCAGATTGTACTGAGAGTGCACC) and MSH2_del_R (CCTTCACTTTTCTAATCCACTCTTTCAGTAAAGCCTTCAAACGAACGCATCTGTGCGGTATTTCACACCGC). The same strategy was used for deletion of the *PMS1* and *MLH1* gene. To obtain pms1Δ we used oligos PMS1_A (TATCAAAGCTAGATCATATTTCGTAATCCTTCGAAAATGAGCTCCAATCACGTAAAATATCTTGACCGCAGTTAA) and PMS1_S (AAGGTGTAAGCAAAAGGAACAGAGGTATATCCCTGTGAAATATTTATTTAGCCCCTATGAACATATTCCATT). The *mlh1Δ* was obtained by transformation with the PCR product obtained from the pRS306 plasmid using oligos MLH1_A (AAGTTAACACCTCTCAAAAACTTTGTATAGATCTGGAAGGTTGGCTATTTCCAACACCGCAGGGTAATAACTGAT) and MLH1_S (ATACGATAGTGATAGTAAATGGAAGGTAAAAATAACATAGACCTATCAATAAGCACGGTCACAGCTTGTCTGTAA).

### Measurement of spontaneous mutation rates

The fluctuation tests to determine spontaneous mutation rates were, unless otherwise indicated, performed in two to five independent experiments of nine independent cultures each with independently obtained derivatives. Single two-day-old colonies from YPD plates were inoculated in 5 ml of liquid YPD medium and were grown with strong aeration for two days and processed as described [33].

### Sequencing of His^+^ revertants and Can^r^ mutants

Independent His^+^ revertants and Can^r^ mutants were grown as small patches on YPD plates. Regions of corresponding genes were amplified by PCR. Amplified DNAs were purified by QIAGEN PCR purification kit and sequenced by MWG Biotech (www.mwgdna.com). *CAN1* spectra obtained in four strains were compared using several statistical techniques. A Monte Carlo modification of the Pearson **χ^2^** test of spectra homogeneity [69] was used to compare 2 x N tables (two mutation spectra, N≥2). Small probability values (P≤0.05) indicate a significant difference between two spectra. Calculations were done using the program COLLAPSE [70].

### Purification of Pol ε, Pol2/Dpb2, and Pol2/Dpb2 *exo-* complexes

All purification steps were carried out as described in [32].

### 3′→5′exonuclease processivity and primer extension assay

We used primer 3NY (5′-AGGTCACGATGCGGCATAGCCTGCATTGATCGCACGATGATCAGCGGACTGCTTACC) annealed to the template 19wt (3′-TCCAGTGCTACGCCGTATCGGACGTAACTAGCGTGCTACTAGTCGCCTGACGAATGGACAGTGCCATTGTCACTG) as a substrate for the exonuclease reaction. The primer (8 µM) was labeled with 40 µCi of [γ-^32^P]ATP in a 20-µl reaction with 10 U T4 polynucleotide kinase (Promega) for 1 h. The reaction was stopped with EDTA and labeled products were purified through PAGE. The end-labeled primer was annealed to the template at 1.5: 1 ratio for a 5 minute incubation at 80°C followed by slow cooling to room temperature. For the exonuclease assay 0.5 nM substrate was incubated with 0.1 nM Pol ε or 0.5 nM Pol2/Dpb2 complex in a 65 µl reaction mixture (40 mM Tris-HCl pH 7.8; 1 mM DTT; 0.2 mg/ml Ac-BSA; 8 mM MgCl_2_; 125 mM NaAc). Fifteen µl aliquots were taken at the indicated time points and were mixed with 8 µl stop solution (80% formamide; 50 mM EDTA; 1 mM bromophenol blue). Before loading the reactions on a 12% polyacrylamide-urea gel, the primer-templates were denatured at 99° C for 4 min and then cooled on ice. The intensity of bands corresponding to different exonuclease products was quantified using phosphoimager plates and the ImageQuant software package supplied with a Typhoon 9400 phosphoimager (Amersham Biosciences).

For primer extension assay a [γ-^32^P]ATP –labeled (as described previously) 50-mer oligonucleotide was annealed to the pBluescript II SK(+) ssDNA in a ratio of 1∶1.5. For the DNA synthesis processivity assay, the substrate (14 nM) was incubated with the four-subunit Pol ε (0.35 nM) or Pol2/Dpb2 complex (0.7 nM) in a reaction mixture (40 mM Tris-HCl pH 7.8; 1 mM DTT; 0.2 mg/ml Ac-BSA; 8 mM MgCl2; 125 mM NaAc; 100 µM dNTP). Because the loading efficiency of the Pol2/Dpb2 complex on DNA was compromised we used a two and five-fold higher concentration of the Pol2/Dpb2 complex, compared to the full-subunit Pol ε, in the primer-extension assay and exonuclease assay, respectively. The conditions were empirically determined to meet single-hit kinetics, i.e. where a polymerase molecule never re-associated with a previously extended primer.

In the primer-extension assay, the termination probability at position N at each primer/template was calculated by dividing the intensity of the band N by the intensity of all bands ≥ N. In the exonuclease processivity assay, the termination probability at position N at each primer/template was calculated by dividing the intensity of the band N by the intensity of all bands ≤ N [71].

### Assay to measure polymerase fidelity *in vitro*

DNA synthesis fidelity was measured using the bacteriophage M13mp2 forward mutation assay described previously [39], [40]. Briefly, double-stranded M13mp2 DNA with a 407-nucleotide single-stranded region containing a portion of the *lacZ* gene was used as a substrate for *in vitro* DNA synthesis. Reactions mixtures contained ∼1.5 nM DNA template, 50 mM Tris-Cl (pH 7.5), 2 mM DTT, 100 µg/ml BSA, 10% glycerol, 250 µM dNTPs and 14 nM wild type or exonuclease-deficient 2-subunit Pol ε. Reactions were incubated at 30°C for 30 min. Aliquots of the reactions were analyzed by agarose gel electrophoresis to confirm complete gap-filling, and another aliquot of DNA was introduced into *E.coli* to score the frequency of light blue and colorless plaques reflecting errors made during *in vitro* DNA synthesis. Single stranded DNA was isolated from independent mutant M13 plaques and the *lacZ* gene was sequenced. Error rates (ER) for individual types of mutation were calculated according to the following equation: ER  =  [(N_i_/N)×MF]/(D×0.6) where N_i_ is the number of mutations of a particular type, N is the total number of mutants analyzed, MF is frequency of *lacZ* mutants, D is the number of detectable sites for the particular type of mutation, and 0.6 is the probability of expressing a mutant *lacZ* allele in *E. coli*.

## Supporting Information

Table S1 Probability that two mutation spectra from *CAN1* gene are homogeneous. (*) indicates that two spectra are different. Raw numbers from Table 5 were used as input. Statistical analysis was performed using the COLLAPSE program [66].
(0.03 MB DOC)

Table S2 Mutations found in *CAN1* when sequencing Canr alleles.
(0.34 MB DOC)

## References

[1] Bebenek K, Kunkel TA (2004). Functions of DNA polymerases.. Adv Protein Chem.

[2] Garg P, Burgers PM (2005). DNA polymerases that propagate the eukaryotic DNA replication fork.. Crit Rev Biochem Mol Biol.

[3] Jin YH, Obert R, Burgers PM, Kunkel TA, Resnick MA (2001). The 3′–>5′ exonuclease of DNA polymerase delta can substitute for the 5′ flap endonuclease Rad27/Fen1 in processing Okazaki fragments and preventing genome instability.. Proc Natl Acad Sci U S A.

[4] Jin YH, Ayyagari R, Resnick MA, Gordenin DA, Burgers PM (2003). Okazaki fragment maturation in yeast. II. Cooperation between the polymerase and 3′-5′-exonuclease activities of Pol delta in the creation of a ligatable nick.. J Biol Chem.

[5] Garg P, Stith CM, Sabouri N, Johansson E, Burgers PM (2004). Idling by DNA polymerase delta maintains a ligatable nick during lagging-strand DNA replication.. Genes Dev.

[6] Nick McElhinny SA, Gordenin DA, Stith CM, Burgers PM, Kunkel TA (2008). Division of labor at the eukaryotic replication fork.. Mol Cell.

[7] Pursell ZF, Isoz I, Lundstrom EB, Johansson E, Kunkel TA (2007). Yeast DNA polymerase epsilon participates in leading-strand DNA replication.. Science.

[8] Shcherbakova PV, Pavlov YI (1996). 3′–>5′ exonucleases of DNA polymerases epsilon and delta correct base analog induced DNA replication errors on opposite DNA strands in Saccharomyces cerevisiae.. Genetics.

[9] Morrison A, Sugino A (1994). The 3′–>5′ exonucleases of both DNA polymerases delta and epsilon participate in correcting errors of DNA replication in Saccharomyces cerevisiae.. Mol Gen Genet.

[10] Karthikeyan R, Vonarx EJ, Straffon AF, Simon M, Faye G (2000). Evidence from mutational specificity studies that yeast DNA polymerases delta and epsilon replicate different DNA strands at an intracellular replication fork.. J Mol Biol.

[11] Pavlov YI, Frahm C, Nick McElhinny SA, Niimi A, Suzuki M (2006). Evidence that errors made by DNA polymerase alpha are corrected by DNA polymerase delta.. Curr Biol.

[12] Pavlov YI, Shcherbakova PV (2010). DNA polymerases at the eukaryotic fork-20 years later.. Mutat Res.

[13] Kunkel TA, Burgers PM (2008). Dividing the workload at a eukaryotic replication fork.. Trends Cell Biol.

[14] Burgers PM (2008). Polymerase dynamics at the eukaryotic DNA replication fork.. J Biol Chem.

[15] Budzowska M, Kanaar R (2009). Mechanisms of dealing with DNA damage-induced replication problems.. Cell Biochem Biophys.

[16] Lopes M, Foiani M, Sogo JM (2006). Multiple mechanisms control chromosome integrity after replication fork uncoupling and restart at irreparable UV lesions.. Mol Cell.

[17] Karras GI, Jentsch S (2010). The RAD6 DNA damage tolerance pathway operates uncoupled from the replication fork and is functional beyond S phase.. Cell.

[18] Daigaku Y, Davies AA, Ulrich HD (2010). Ubiquitin-dependent DNA damage bypass is separable from genome replication.. Nature.

[19] Pomerantz RT, O'Donnell M (2008). The replisome uses mRNA as a primer after colliding with RNA polymerase.. Nature.

[20] Chilkova O, Jonsson BH, Johansson E (2003). The quaternary structure of DNA polymerase epsilon from Saccharomyces cerevisiae.. J Biol Chem.

[21] Araki H, Hamatake RK, Johnston LH, Sugino A (1991). DPB2, the gene encoding DNA polymerase II subunit B, is required for chromosome replication in Saccharomyces cerevisiae.. Proc Natl Acad Sci U S A.

[22] Feng W, Rodriguez-Menocal L, Tolun G, D'Urso G (2003). Schizosacchromyces pombe Dpb2 binds to origin DNA early in S phase and is required for chromosomal DNA replication.. Mol Biol Cell.

[23] Kesti T, McDonald WH, Yates JR, Wittenberg C (2004). Cell cycle-dependent phosphorylation of the DNA polymerase epsilon subunit, Dpb2, by the Cdc28 cyclin-dependent protein kinase.. J Biol Chem.

[24] Jaszczur M, Flis K, Rudzka J, Kraszewska J, Budd ME (2008). Dpb2p, a noncatalytic subunit of DNA polymerase epsilon, contributes to the fidelity of DNA replication in Saccharomyces cerevisiae.. Genetics.

[25] Jaszczur M, Rudzka J, Kraszewska J, Flis K, Polaczek P (2009). Defective interaction between Pol2p and Dpb2p, subunits of DNA polymerase epsilon, contributes to a mutator phenotype in Saccharomyces cerevisiae.. Mutat Res.

[26] Araki H, Hamatake RK, Morrison A, Johnson AL, Johnston LH (1991). Cloning DPB3, the gene encoding the third subunit of DNA polymerase II of Saccharomyces cerevisiae.. Nucleic Acids Res.

[27] Northam MR, Garg P, Baitin DM, Burgers PM, Shcherbakova PV (2006). A novel function of DNA polymerase zeta regulated by PCNA.. EMBO J.

[28] Li Y, Pursell ZF, Linn S (2000). Identification and cloning of two histone fold motif-containing subunits of HeLa DNA polymerase epsilon.. J Biol Chem.

[29] Tsubota T, Maki S, Kubota H, Sugino A, Maki H (2003). Double-stranded DNA binding properties of Saccharomyces cerevisiae DNA polymerase epsilon and of the Dpb3p-Dpb4p subassembly.. Genes Cells.

[30] Iida T, Araki H (2004). Noncompetitive counteractions of DNA polymerase epsilon and ISW2/yCHRAC for epigenetic inheritance of telomere position effect in Saccharomyces cerevisiae.. Mol Cell Biol.

[31] Tackett AJ, Dilworth DJ, Davey MJ, O'Donnell M, Aitchison JD (2005). Proteomic and genomic characterization of chromatin complexes at a boundary.. J Cell Biol.

[32] Asturias FJ, Cheung IK, Sabouri N, Chilkova O, Wepplo D (2006). Structure of Saccharomyces cerevisiae DNA polymerase epsilon by cryo-electron microscopy.. Nat Struct Mol Biol.

[33] Shcherbakova PV, Kunkel TA (1999). Mutator phenotypes conferred by MLH1 overexpression and by heterozygosity for mlh1 mutations.. Mol Cell Biol.

[34] Tran HT, Keen JD, Kricker M, Resnick MA, Gordenin DA (1997). Hypermutability of homonucleotide runs in mismatch repair and DNA polymerase proofreading yeast mutants.. Mol Cell Biol.

[35] Tran HT, Gordenin DA, Resnick MA (1999). The 3′–>5′ exonucleases of DNA polymerases delta and epsilon and the 5′–>3′ exonuclease Exo1 have major roles in postreplication mutation avoidance in Saccharomyces cerevisiae.. Mol Cell Biol.

[36] Kirchner JM, Tran H, Resnick MA (2000). A DNA polymerase epsilon mutant that specifically causes +1 frameshift mutations within homonucleotide runs in yeast.. Genetics.

[37] Dang W, Kagalwala MN, Bartholomew B (2007). The Dpb4 subunit of ISW2 is anchored to extranucleosomal DNA.. J Biol Chem.

[38] Gangaraju VK, Prasad P, Srour A, Kagalwala MN, Bartholomew B (2009). Conformational changes associated with template commitment in ATP-dependent chromatin remodeling by ISW2.. Mol Cell.

[39] Bebenek K, Kunkel TA (1995). Analyzing fidelity of DNA polymerases.. Methods Enzymol.

[40] Shcherbakova PV, Pavlov YI, Chilkova O, Rogozin IB, Johansson E (2003). Unique error signature of the four-subunit yeast DNA polymerase epsilon.. J Biol Chem.

[41] Lawrence CW (2004). Cellular functions of DNA polymerase zeta and Rev1 protein.. Adv Protein Chem.

[42] Northam MR, Robinson HA, Kochenova OV, Shcherbakova PV (2009). Participation of DNA Polymerase {zeta} in Replication of Undamaged DNA in Saccharomyces cerevisiae.. Genetics.

[43] Zhong X, Garg P, Stith CM, Nick McElhinny SA, Kissling GE (2006). The fidelity of DNA synthesis by yeast DNA polymerase zeta alone and with accessory proteins.. Nucleic Acids Res.

[44] Pavlov YI, Shcherbakova PV, Kunkel TA (2001). In vivo consequences of putative active site mutations in yeast DNA polymerases alpha, epsilon, delta, and zeta.. Genetics.

[45] Harfe BD, Jinks-Robertson S (2000). DNA mismatch repair and genetic instability.. Annu Rev Genet.

[46] Tran HT, Degtyareva NP, Gordenin DA, Resnick MA (1999). Genetic factors affecting the impact of DNA polymerase delta proofreading activity on mutation avoidance in yeast.. Genetics.

[47] Huang ME, Rio AG, Galibert MD, Galibert F (2002). Pol32, a subunit of Saccharomyces cerevisiae DNA polymerase delta, suppresses genomic deletions and is involved in the mutagenic bypass pathway.. Genetics.

[48] Chilkova O, Stenlund P, Isoz I, Stith CM, Grabowski P (2007). The eukaryotic leading and lagging strand DNA polymerases are loaded onto primer-ends via separate mechanisms but have comparable processivity in the presence of PCNA.. Nucleic Acids Res.

[49] Kokoska RJ, Stefanovic L, DeMai J, Petes TD (2000). Increased rates of genomic deletions generated by mutations in the yeast gene encoding DNA polymerase delta or by decreases in the cellular levels of DNA polymerase delta.. Mol Cell Biol.

[50] Xie Y, Counter C, Alani E (1999). Characterization of the repeat-tract instability and mutator phenotypes conferred by a Tn3 insertion in RFC1, the large subunit of the yeast clamp loader.. Genetics.

[51] Niimi A, Limsirichaikul S, Yoshida S, Iwai S, Masutani C (2004). Palm mutants in DNA polymerases alpha and eta alter DNA replication fidelity and translesion activity.. Mol Cell Biol.

[52] Chen C, Umezu K, Kolodner RD (1998). Chromosomal rearrangements occur in S. cerevisiae rfa1 mutator mutants due to mutagenic lesions processed by double-strand-break repair.. Mol Cell.

[53] Yang Y, Sterling J, Storici F, Resnick MA, Gordenin DA (2008). Hypermutability of damaged single-strand DNA formed at double-strand breaks and uncapped telomeres in yeast Saccharomyces cerevisiae.. PLoS Genet.

[54] Jin YH, Garg P, Stith CM, Al-Refai H, Sterling JF (2005). The multiple biological roles of the 3′–>5′ exonuclease of Saccharomyces cerevisiae DNA polymerase delta require switching between the polymerase and exonuclease domains.. Mol Cell Biol.

[55] Swan MK, Johnson RE, Prakash L, Prakash S, Aggarwal AK (2009). Structural basis of high-fidelity DNA synthesis by yeast DNA polymerase delta.. Nat Struct Mol Biol.

[56] Gutierrez PJ, Wang TS (2003). Genomic instability induced by mutations in Saccharomyces cerevisiae POL1.. Genetics.

[57] Tran HT, Gordenin DA, Resnick MA (1996). The prevention of repeat-associated deletions in Saccharomyces cerevisiae by mismatch repair depends on size and origin of deletions.. Genetics.

[58] Kunkel TA, Erie DA (2005). DNA mismatch repair.. Annu Rev Biochem.

[59] Mojas N, Lopes M, Jiricny J (2007). Mismatch repair-dependent processing of methylation damage gives rise to persistent single-stranded gaps in newly replicated DNA.. Genes Dev.

[60] Lehner K, Jinks-Robertson S (2009). The mismatch repair system promotes DNA polymerase zeta-dependent translesion synthesis in yeast.. Proc Natl Acad Sci U S A.

[61] Delbos F, Aoufouchi S, Faili A, Weill JC, Reynaud CA (2007). DNA polymerase eta is the sole contributor of A/T modifications during immunoglobulin gene hypermutation in the mouse.. J Exp Med.

[62] Albertson TM, Ogawa M, Bugni JM, Hays LE, Chen Y (2009). DNA polymerase {varepsilon} and {delta} proofreading suppress discrete mutator and cancer phenotypes in mice.. Proc Natl Acad Sci U S A.

[63] Goldstein AL, McCusker JH (1999). Three new dominant drug resistance cassettes for gene disruption in Saccharomyces cerevisiae.. Yeast.

[64] Pavlov YI, Maki S, Maki H, Kunkel TA (2004). Evidence for interplay among yeast replicative DNA polymerases alpha, delta and epsilon from studies of exonuclease and polymerase active site mutations.. BMC Biol.

[65] Morrison A, Bell JB, Kunkel TA, Sugino A (1991). Eukaryotic DNA polymerase amino acid sequence required for 3′––5′ exonuclease activity.. Proc Natl Acad Sci U S A.

[66] Jin YH, Obert R, Burgers PM, Kunkel TA, Resnick MA (2001). The 3′–>5′ exonuclease of DNA polymerase delta can substitute for the 5′ flap endonuclease Rad27/Fen1 in processing Okazaki fragments and preventing genome instability.. Proc Natl Acad Sci U S A.

[67] Pavlov YI, Mian IM, Kunkel TA (2003). Evidence for preferential mismatch repair of lagging strand DNA replication errors in yeast.. Curr Biol.

[68] Shcherbakova PV, Noskov VN, Pshenichnov MR, Pavlov YI (1996). Base analog 6-N-hydroxylaminopurine mutagenesis in the yeast Saccharomyces cerevisiae is controlled by replicative DNA polymerases.. Mutat Res.

[69] Adams WT, Skopek TR (1987). Statistical test for the comparison of samples from mutational spectra.. J Mol Biol.

[70] Khromov-Borisov NN, Rogozin IB, Pegas Henriques JA, de Serres FJ (1999). Similarity pattern analysis in mutational distributions.. Mutat Res.

[71] Kokoska RJ, McCulloch SD, Kunkel TA (2003). The efficiency and specificity of apurinic/apyrimidinic site bypass by human DNA polymerase eta and Sulfolobus solfataricus Dpo4.. J Biol Chem.

