# Supplementary material for: Mismatch Repair–Independent Increase in Spontaneous Mutagenesis in Yeast Lacking Non-Essential Subunits of DNA Polymerase ε
Source: PLoS Genet. 2010 Nov 18;6(11):e1001209. doi: 10.1371/journal.pgen.1001209 (PMC2987839; doi:10.1371/journal.pgen.1001209)
Supplement: Table S1 — Probability that two mutation spectra from CAN1 gene are homogeneous. (*) indicates that two spectra are different. Raw numbers from Table 5 were used as input. Statistical analysis was performed using the COLLAPSE program [66]. (0.03 MB DOC) [file pgen.1001209.s001.doc]

| Strain | *dpb3Δ dpb4Δ* | *pol2-4* | *dpb3Δ dpb4Δ pol2-4* |
| --- | --- | --- | --- |
| Wild type | 0.72 | 0.007* | 0.0007* |
| *dpb3Δ dpb4Δ* |  | 0.003* | 0.016* |
| *pol2-4* |  |  | 0.074 |
