# Supplementary material for: Mismatch Repair–Independent Increase in Spontaneous Mutagenesis in Yeast Lacking Non-Essential Subunits of DNA Polymerase ε
Source: PLoS Genet. 2010 Nov 18;6(11):e1001209. doi: 10.1371/journal.pgen.1001209 (PMC2987839; doi:10.1371/journal.pgen.1001209)
Supplement: Table S2 — Mutations found in CAN1 when sequencing Canr alleles. (0.34 MB DOC) [file pgen.1001209.s002.doc]

Table S2. **Mutations found in *CAN1* when sequencing *Can^r^* alleles**

***Wild-type E134***

| **mutant** | **position** | **wt** | **mutation** | **wt sequence** | **mutated sequence** |
| --- | --- | --- | --- | --- | --- |
| 1 | 532 | G | C | TTGG**g**CAAT | TTGG**c**CAAT |
| 2 | 285 | G | A | GTAT**g**ATTG | GTAT**a**ATTG |
| 3 | 1517 | T | G | TTTA**t**GACG | TTTA**g**GACG |
| 4 | 365-435 |  | deletion |  |  |
| 5 | 352 | G | T | CGCC**g**GCCC | CGCC**t**GCCC |
| 6 | 311 | C | T | GGTA**c**AGGT | GGTA**t**AGGT |
| 7 | 1493-1551 |  | deletion |  |  |
| 8 | 113 | C | G | GGGT**c**AATA | GGGT**g**AATA |
| 9 | 887 | C | G | TTCA**c**ATTT | TTCA**g**ATTT |
| 10 | 691 | G | C | GGTC**g**CTTC | GGTC**c**CTTC |
| 11 | 1208 | G | A | TTTG**g**TCTA | TTTG**a**TCTA |
| 12 | 1244 | C | A | CTGT**c**AAGG | CTGT**a**AAGG |
| 13 | 393-450 |  | deletion |  |  |
| 14 | 113 | C | G | GGGT**c**AATA | GGGT**g**AATA |
| 15 | 626 |  | +1 insertion T | TTTTTT-gGG | TTTTTT**tc**GG |
|  | 627 | G | C | TTTTTT-gGG | TTTTTT**tc**GG |
| 16 | 1214 | C | G | CTAT**c**AAAG | CTAT**g**AAAG |
| 17 | 1163 | C | A | ATTT**c**TGCC | ATTT**a**TGCC |
| 18 | 530 | G | A | TCTT**g**GGCA | TCTT**a**GGCA |
| 19 | 1450-1644 |  | deletion |  |  |
| 20 | 768 | C | -1 deletion | GG**c**CCAG | CCGG**-**CCAG |
| 21 | 509-559 |  | deletion |  |  |
| 22 | 442 | C | G | CATC**c**CTGT | CATC**g**CTGT |
| 23 | 1035 | C | G | CATA**c**AATG | CATA**g**AATG |
| 24 | 669 | C | A | ATTA**c**GGTG | ATTA**a**GGTG |
| 25 | 959 | C | A | AAGAG**C**CAT | AAGAG**A**CAT |
| 26 | 887 | C | G | TTCA**c**ATTT | TTCA**g**ATTT |
| 27 | 674 | A | C | GGTG**a**ATTC | GGTG**c**ATTC |
| 28 | 1268 | C | G | GTTC**c**ATAC | GTTC**g**ATAC |
| 29 | 1196 | G | C | TCCC**g**TATT | TCCC**c**TATT |
| 30 | 355 | C | A | CGGC**c**CAGT | CGGC**a**CAGT |
| 31 | 768 | C | -1 deletion | GG**c**CCAG | CCGG**-**CCAG |
| 32 | 740 |  | +1 insertion T | TTTTT**-**GT | TTTTT**t**GT |
| 33 | 257 | G | C | AAGA**g**AGAG | AAGA**c**AGAG |
| 34 | 1163 | C | T | ATTT**c**TGCC | ATTT**t**TGCC |
| 35 | 1255 | A | T | CACC**a**AAGG | CACC**t**AAGG |
| 36 | 526 | T | C | GTTT**t**CTTG | GTTT**c**CTTG |
| 37 | 415 | T | C | GCAG**t**CCTT | GCAG**c**CCTT |
| 38 | 314 | G | T | ACAG**g**TCTT | ACAG**t**TCTT |
| 39 | 838 | G | T | AAAC**g**AAGG | AAAC**t**AAGG |
| 40 | 527 | C | T | TTTT**c**TTGG | TTTT**t**TTGG |
| 41 | 424 | G | A | GGGT**g**AAAT | GGGT**a**AAAT |
| 42 | 884 | T | C | GCCT**t**CACA | GCCT**c**CACA |
| 43 | 353 | G | T | GCCG**g**CCCA | GCCG**t**CCCA |
| 44 | 353 | G | T | GCCG**g**CCCA | GCCG**t**CCCA |
| 45 | 1227 | G | -1 deletion | TT**g**GCT | TT**-**GCT |
| 46 | 1558 | C | -1 deletion | CA**c**CAAAA | CA**-**CAAAA |
| 47 | 686 | G | -1 deletion | CT**g**GGTC | CT**-**GGTC |
| 48 | 620 |  | +1 insertion T | TA-TTTTTTGG | TA**t**TTTTTTGG |

***Δdpb3 Δdpb4***

| **mutant** | **position** | **wt** | **mutation** | **wt sequence** | **mutated sequence** |
| --- | --- | --- | --- | --- | --- |
| 1 | 1413 | C | -1 deletion | GCA**c**ATCA | GCA**-**ATCA |
| 2 | 1397-1400 |  | deletion TTAT | TTAT**ttat**CTC | TTAT----CTC |
| 3 | 1018 | G | A | CATT**g**GACT | CATT**a**GACT |
| 4 | 911 | G | T | GTTG**g**TATC | GTTG**t**TATC |
| 5 | 620 | T | -1 deletion | TA**t**TTTTTG | TA-TTTTTG |
| 6 | 1022 | T | C | GGAC**t**TTTA | GGAC**c**TTTA |
| 7 | 686 | G | A | TTCT**g**GGTC | TTCT**a**GGTC |
| 8 | 430 | G | C | AATG**g**CTAC | AATG**c**CTAC |
| 9 | 1486 | C | G | AATG**c**CCGG | AATG**g**CCGG |
| 10 | 1370 | G | A | ACTG**g**TGTT | ACTG**a**TGTT |
| 11 | 1151 | C | G | TTAA**c**AACC | TTAA**g**AACC |
| 12 | 387 | G | -1 deletion | AT**g**GGTTCT | AT-GGTTCT |
|  | 602 | T | A | CCAC**t**GGCG | CCAC**a**GGCG |
| 13 | 938 | C | T | AACC**c**CAGA | AACC**t**CAGA |
| 14 | 1487 | C | T | ATGC**c**CGGC | ATGC**t**CGGC |
| 15 | 311 | C | G | GGTA**c**AGGT | GGTA**g**AGGT |
| 16 | 1123 | C | -1 deletion | TTTG**c**CACA | TTTG**-**CACA |
| 17 | 1082 | C | G | TCTC**c**CTTT | TCTC**g**CTTT |
| 18 | 442 | C | T | CATC**c**CTGT | CATC**t**CTGT |
| 19 | 903-955 |  | deletion |  |  |
| 20 | 620 | T | -1 deletion | TA**t**TTTTTGG | TA-TTTTTGG |
| 21 | 591 | C | G | CGTA**c**AAAG | CGTA**g**AAAG |
| 22 | 591 | C | G | CGTA**c**AAAG | CGTA**g**AAAG |
| 23 | 670 | G | C | TTAC**g**GTGA | TTAC**c**GTGA |
| 24 | 1211 | T | C | TC**t**A**t**CAAA | TC**c**A**c**CAAA |
|  | 1213 | T | C | TC**t**A**t**CAAA | TC**c**A**c**CAAA |
| 25 | 1373 | T | -1 deletion | GTG**t**TGCAG | GTG**-**TGCAG |
| 26 | 728 | T | G | CTAA**t**ATAC | CTAA**g**ATAC |
| 27 | 580 | T | -1 deletion | TCAA**t**TTTG | TCAA**-**TTTG |
| 28 | 416 | C | A | CAGT**c**CTTG | CAGT**a**CTTG |
| 29 | 718 | G | C | TATC**g**GGTT | TATC**c**GGTT |
| 30 | 979 | C | T | TTTC**c**GTAT | TTTC**t**GTAT |
| 31 | 396 | G | -1 deletion | CTTT**g**GCAT | CTTT**-**GCAT |
| 32 | 841 | G | -1 deletion | CGAA**g**GGAG | CGAA**-**GGAG |
| 33 | 150 | T | A | TGTA**t**CCAT | TGTA**a**CCAT |
| 34 | 865 | T | C | CC**t**CTTT**g-**AT | CC**c**CTTT**tt**AT |
|  | 870 | G | T | CC**t**CTTT**g-**AT | TTCC**c**CTTT**tt**ATTA |
|  | 871 |  | +1 insertion T | CC**t**CTTT**g-**AT | CC**c**CTTT**tt**AT |
| 35 | 459 |  | +1 insertion T | CTTT**-c**ACA | CTTT**tg**ACA |
|  | 460 | C | G | CTTT**-c**ACA | CTTT**tg**ACA |
| 36 | 1094 | C | A | ATTG**c**TATT | ATTG**a**TATT |
| 37 | 1099 | G | T | TATT**g**AGAA | TATT**t**AGAA |
| 38 | 1459 | G | T | TGAC**g**AGTT | TGAC**t**AGTT |
| 39 | 1031 | C | T | GTTC**c**ATAC | GTTC**t**ATAC |
| 40 | 787 | T | C | TTAT**t**GGAG | TTAT**c**GGAG |
| 41 | 111 |  | +1 insertion T | GTGG**-**GTCA | GTGG**t**GTCA |
| 42 | 858 | G | A | GTTG**g**GTTT | GTTG**a**GTTT |
| 43 | 1232 | C | T | TC**c**T**a**AATT | TC**c**T**-**AATT |
|  | 1234 | A | -1 deletion | TC**c**T**a**AATT | TC**c**T**-**AATT |
| 44 | 775 | G | T | AGTT**g**GATT | AGTT**t**GATT |
| 45 | 130 | G | T | AGAT**g**AGAA | AGAT**t**AGAA |
| 46 | 1205 | T | C | TTAT**t**TGGT | TTAT**c**TGGT |
| 47 | 1290-1302 |  | complex +1 insertion | TTAC**tg**CT**g**C**a-**  TTTGG**c**GCTT | TTAC**ct**CT**t**C**ct**  TTTGG**g**GCTT |
| 48 | 308 | G | T | ATTG**g**TACA | ATTG**t**TACA |

***pol2-4***

| **mutant** | **position** | **wt** | **mutation** | **wt sequence** | **mutated sequence** |
| --- | --- | --- | --- | --- | --- |
| 1 | 809 | G | A | TGGG**g**TCCA | TGGG**a**TCCA |
| 2 | 1022 |  | +1 insertion T | GGAC-TTTT | GGAC**t**TTTT |
| 3 | 980 | G | T | TTCC**g**TATC | TTCC**t**TATC |
| 4 | 554 | T | G | GAAC**t**TAGT | GAAC**g**TAGT |
| 5 | 374 | C | A | ATAT**c**ATAT | ATAT**a**ATAT |
| 6 | 554 | T | G | GAAC**t**TAGT | GAAC**g**TAGT |
| 7 | 258 | A | C | AGAG**a**GAGC | AGAG**c**GAGC |
| 8 | 905 | T | C | GAAC**t**AGTT | GAAC**c**AGTT |
| 9 | 768 | C | -1 deletion | CGG**c**CCAGT | CGG-CCAGT |
| 10 | 980 | G | A | TTCC**g**TATC | TTCC**a**TATC |
| 11 | 263 | T | A | GAGC**t**TAAG | GAGC**a**TAAG |
| 12 | 929 | C | A | GAAG**c**TGCA | GAAG**a**TGCA |
| 13 | 646 | A | T | AATG**a**ACTT | AATG**t**ACTT |
| 14 | 980 | G | C | TTCC**g**TATC | TTCC**c**TATC |
| 15 | 1214 | C | G | CTAT**c**AAAG | CTAT**g**AAAG |
| 16 | 317 | T | G | GGTC**t**TTTC | GGTC**g**TTTC |
| 17 | 1391 | G | -1 deletion | GCAT**g**GTTA | GCAT-GTTA |
| 18 | 313 | G | T | TACA**g**GTCT | TACA**t**GTCT |
| 19 | 325 | G | -1 deletion | CATT**g**GTTT | CATT-GTTT |
| 20 | 591 | C | A | CGTA**c**AAAG | CGTA**a**AAAG |
| 21 | 127 |  | +1 insertion A | AAAA**-g**ATG | AAAA**ac**ATG |
|  | 128 | G | C | AAAA**-g**ATG | AAAA**ac**ATG |
| 22 | 1128-1152 | deletion |  |  |  |
| 23 | 1343 |  | +1 insertion T | AAAG-TTTT | AAAG**t**TTTT |
| 24 | 620 |  | +1 insertion T | TA-TTTTTT | TA**t**TTTTTT |
| 25 | 664 | T | G | CAAA**t**ATTA | CAAA**g**ATTA |
| 26 | 832 |  | +1 insertion A | GAT-AAAAA | GAT**a**AAAAA |
| 27 | 425 | A | G | GGTG**a**AATG | GGTG**g**AATG |
| 28 | 1381 |  | +1 insertion T | GC-TTTTTT | GC**t**TTTTTT |
| 29 | 475 | A | T | ACAA**a**GATT | ACAA**t**GATT |
| 30 | 1379 | G | A | GCAG**g**CTTT | GCAG**a**CTTT |
| 31 | 1214 | C | G | CTAT**c**AAAG | CTAT**g**AAAG |
| 32 | 735 | T | A | ACTG**t**TTTT | ACTG**a**TTTT |
| 33 | 317 | T | A | GGTC**t**TTTC | GGTC**a**TTTC |
| 34 | 964 |  | +1 insertion A | TC-AAAAAA | TC**a**AAAAAA |
| 35 | 620 |  | +1 insertion T | TA-TTTTTT | TA**t**TTTTTT |
| 36 | 272 | G | T | CAAA**g**ACAT | CAAA**t**ACAT |
| 37 | 970 | G | A | AAAAAA**g-**TT | AAAAAA**at**TT |
|  | 971 |  | +1 insertion T | AAAAAA**g-**TT | AAAAAA**at**TT |
| 38 | 482 | T | C | TTCC**t**TTCT | TTCC**c**TTCT |
| 39 | 317 |  | +1 insertion T | TC-TTTTCA | TC**t**TTTTCA |
| 40 | 1538 | A | G | ATTC**a**AGGT | ATTC**g**AGGT |
| 41 | 1500 | T | A | CTTA**t**TATG | CTTA**a**TATG |
| 42 | 1048 | C | -1 deletion | TAAA**c**TAAC | TAAA**-**TAAC |
| 43 | 832 |  | +1 insertion A | AT-AAAAAC | AT**a**AAAAAC |
| 44 | 735 |  | +1 insertion T | TG-TTTTTG | TG**t**TTTTTG |
| 45 | 700 | A | G | CATC**a**AAGT | CATC**g**AAGT |
| 46 | 655 | C | G | GTTC**c**CTGT | GTTC**g**CTGT |
| 47 | 832 |  | +1 insertion A | AT-AAAAAC | AT**a**AAAAAC |
| 48 | 620 | T | -1 deletion | TA**t**TTTTTG | TA-TTTTTG |

***pol2-4Δdpb3 Δdpb4***

| **mutant** | **position** | **wt** | **mutation** | **wt sequence** | **mutated sequence** |
| --- | --- | --- | --- | --- | --- |
| 1 | 929 | G | C | TGAA**g**CTGC | TGAA**c**CTGC |
| 2 | 355 | C | A | CGGC**cca**GT | CGGC**ag-**GT |
|  | 356 | C | G | CGGC**cca**GT | CGGC**ag-**GT |
|  | 357 | A | -1 deletion | CGGC**cca**GT | CGGC**ag-**GT |
| 3 | 1113 |  | +1 insertion A | TAC-AAAGG | TACaAAAGG |
| 4 | 1054 | C | T | AACA**c**AATC | AACA**t**AATC |
| 5 | 1073 |  | +1 insertion T | TTT**-c**TACT | TTT**tg**TACT |
|  | 1074 | C | G | TTT**-c**TACT | TTT**tg**TACT |
| 6 | 1417 | A | T | CATC**a**GATT | CATC**t**GATT |
| 7 | 184 | G | T | TGGC**g**AGGA | TGGC**t**AGGA |
| 8 | 1359 | A | -1 deletion | TATT**a**AATA | TATT-AATA |
| 9 | 287 |  | +1 insertion T | GATT**-**GCCC | GATT**t**GCCC |
| 10 | 1215 | A | -1 deletion | TATC**a**AAGA | TATC-AAGA |
| 11 | 1099 | G | T | TATT**g**AGAA | TATT**t**AGAA |
| 12 | 1151 | C | G | TTAA**c**AACC | TTAA**g**AACC |
| 13 | 39 |  | insertion +6 A | AA**------**GC | AA**aaaaaa**GC |
| 14 | 950 | T | A | TCCG**t**TCCA | TCCG**a**TCCA |
| 15 | 620 |  | +1 insertion T | TA-TTTTTT | TA**t**TTTTTT |
| 16 | 377 | A | -1 deletion | TCAT**a**TTTA | TCAT**-**TTTA |
| 17 | 1560 | A | -1 deletion | CACC**a**AAAT | CACC-AAAT |
| 18 | 1481 | T | G | AAAT**t**AATG | AAAT**g**AATG |
| 19 | 1099 | G | T | TATT**g**AGAA | TATT**t**AGAA |
| 20 | 1171 | A | T | CGCA**a**ATTC | CGCA**t**ATTC |
| 21 | 1151 | C | G | TTAA**c**AACC | TTAA**g**AACC |
| 22 | 1175 | C | T | AATT**c**AAAT | AATT**t**AAAT |
| 23 | 48 | C | G | TGTA**c**AATG | TGTA**g**AATG |
| 24 | 959 | C | A | AGAG**c**CATC | AGAG**a**CATC |
| 25 | 290 | C | A | ATTG**c**CCTT | ATTG**a**CCTT |
| 26 | 1373 | T | G | GGTG**t**TGCA | GGTG**g**TGCA |
| 27 | 661 | A | -1 deletion | TGTC**a**AATA | TGTC-A**A**TA |
| 28 | 877 | G | C | TAAC**g**CTGC | TAAC**c**CTGC |
| 29 | 973-975 | GTT | deletion | GTT**gtt**TTC | GTT**---**TTC |
| 30 | 428 | T | A | GAAA**t**GGCT | GAAA**a**GGCT |
| 31 | 368 | T | G | GCTC**t**TATA | GCTC**g**TATA |
| 32 | 893 | A | C | TTTC**a**AGGT | TTTC**c**AGGT |
| 33 | 735 |  | +1 insertion T | TG-TTTTTG | TGtTTTTTG |
| 34 | 48 | C | A | TGTA**c**AATG | TGTA**a**AATG |
| 35 | 964 | A | -1 deletion | ATC**a**AAAAA | ATC-AAAAA |
| 36 | 535-561 | deletion |  |  |  |
| 37 | 1284 |  | +1 insertion T | TTTT**-c**GTT | TTTT**tg**GTT |
|  | 1285 | C | G | TTTT**-c**GTT | TTTT**tg**GTT |
| 38 | 1435 | A | -1 deletion | TTTG**a**AATA | TTTG-AATA |
| 39 | 648 | C | G | TGAA**c**TTGT | TGAA**g**TTGT |
| 40 | 682 | T | A | CGAG**t**TCTG | CGAG**a**TCTG |
| 41 | 1468 | T | -1 deletion | ACCA**t**TTAA | ACCA-TTAA |
| 42 | 620 | T | +1 insertion | TA-TTTTTT | TA**t**TTTTTT |
| 43 | 658 | G | C | CCCT**g**TCAA | CCCT**c**TCAA |
| 44 | 508 | G | C | CAAT**g**GTTA | CAAT**c**GTTA |
| 45 | 317 |  | +1 insertion T | GGTC-TTTT | GGTCtTTTT |
| 46 | 1391 | G | -1 deletion | GCAT**g**GTTA | GCAT-GTTA |
| 47 | 142 | G | T | TAAA**g**AATT | TAAA**t**AATT |
| 48 | 271 | A | T | GCAA**a**GACA | GCAA**t**GACA |
